# Neural reward-related reactions to monetary gains for self and charity

**DOI:** 10.3758/s13415-018-00672-1

**Published:** 2018-11-28

**Authors:** Jochem P. Spaans, Sabine Peters, Eveline A. Crone

**Affiliations:** 1 Department of Developmental and Educational Psychology, Institute of Psychology, Leiden University, Leiden, The Netherlands; 2 Leiden Institute for Brain and Cognition, Leiden University, Leiden, The Netherlands

**Keywords:** Charity donations, Vicarious gaining, Empathy

## Abstract

The aim of the present study was to examine the neural signatures of gaining money for self and charity. Young adults (*N* = 31, 21–24 years of age) underwent fMRI scanning while they performed a task in which they could earn money for themselves and for a self-chosen charity by selecting one of two options with unknown outcomes. The results showed elevated activity in the ventral striatum when gaining for the self only and for self and charity, but not when gaining for charity only. However, increased ventral striatal activity when gaining for charity only was correlated with participants’ self-reported empathic concern and enjoyment when winning for charity. Empathic concern was also related to donating a larger proportion of earnings to charity after the MRI session. In short, these results reveal robust ventral striatal activity when gaining for oneself, but empathy-dependent individual differences in ventral striatal activity when gaining for charity.

Prosocial behavior is an important social glue within society, leading, for example, to charity donations and helping behavior toward unknown others (Eisenberg, Fabes, & Spinrad, 2006). In addition to its benefits for others, prosocial behavior affects the donator as well. For instance, there is a strong relationship between prosocial behavior and happiness, which is universal across countries and cultures (Aknin et al., 2013), suggesting that acting prosocially feels good. Indeed, researchers who have examined the neural components of prosociality have theorized that this hedonic aspect, “the warm glow of giving,” might be a possible motivator to perform prosocial behavior (Andreoni, 1990; Harbaugh, Mayr, & Burghart, 2007; Moll et al., 2006). In the present study, we aimed to test the processes related to this hedonistic aspect of prosociality.

One way to gain more insight into the rewarding feeling of being prosocial is through studying responses when gaining rewards for others (often referred to as *vicarious gaining*), and through comparing these responses to those observed when gaining rewards for oneself. Gaining for oneself has consistently been associated with activity in the ventral striatum (Báez-Mendoza & Schultz, 2013; Berridge & Kringelbach, 2015; Delgado, 2007; Montague & Berns, 2002; Schultz, 2016; Sescousse, Caldú, Segura, & Dreher, 2013), but there is some controversy regarding this rewarding effect of vicarious gains. That is, several studies have shown that vicarious gaining was associated with activation in the ventral striatum (Mobbs et al., 2009; Telzer, Fuligni, Lieberman, & Galvan, 2013), such as when gaining rewards for friends (Braams, Güroǧlu, et al., 2014a; Varnum, Shi, Chen, Qiu, & Han, 2014), close others (Inagaki et al., 2016), or charities (Harbaugh et al., 2007; Moll et al., 2006). However, this rewarding effect has not been found in all studies (Morelli, Sacchet, & Zaki, 2015). That is, ventral striatal activity during vicarious gaining is likely dependent on individual differences in how important the other is to you (Braams, Peters, Peper, Güroǧlu, & Crone, 2014; Telzer et al., 2013), in willingness to give to others (Kuss et al., 2013), in relationship closeness (Fareri, Niznikiewicz, Lee, & Delgado, 2012), and in traits such as empathic concern (Andreoni, Rao, & Trachtman, 2017), although no prior study has tested this experimentally. One study on vicarious reward prediction has shown that trait empathy was correlated with anterior cingulate cortex activation when predicting vicarious rewards, but not when predicting personal rewards, which supports the hypothesis that trait empathy could be related to aspects of vicarious-reward processing (Lockwood, Apps, Roiser, & Viding, 2015).

Besides the role that the ventral striatum might play in the valuation of personal and vicarious rewards, activity in the VS during personal and vicarious gains might play an integral role in social reward learning (Lockwood, Apps, Valton, Viding, & Roiser, 2016). For instance, Lockwood et al. (2016) used a probabilistic learning task in which participants could earn rewards for themselves and others, and showed that striatal activity during the task was related to prediction errors for rewards, regardless of the beneficiary. Thus, these findings suggest that the ventral striatum might serve a more general role in learning (e.g., irrespective of target).

In addition to the literature on the neural correlates of obtaining personal rewards and vicarious rewards in a vacuum (e.g., in separate conditions), some studies have focused on shared rewards instead. For instance, it was previously found that individuals show stronger ventral striatum activity when both parties cooperate in the prisoner’s dilemma (Krill & Platek, 2012; Rilling, Sanfey, Aronson, Nystrom, & Cohen, 2004), suggesting that shared outcomes are more rewarding than gaining at the expense of others (Güroglu, van den Bos, & Crone, 2014; Rilling et al., 2002). Higher ventral striatum activity has also been found when reciprocating the trust of unknown others, but only when engaging in multiple interactions with the same individuals (Bellucci, Chernyak, Goodyear, Eickhoff, & Krueger, 2017), and for individuals who had more prosocial tendencies (Van den Bos, Van Dijk, Westenberg, Rombouts, & Crone, 2009). In addition, gaining for self at the expense of others’ outcomes was related to more activity in anterior medial prefrontal cortex and anterior cingulate cortex (Feng, Luo, & Krueger, 2015; Van den Bos et al., 2009). The medial prefrontal cortex has previously been associated with a myriad of cognitive processes, amongst which are self-reflection (D’Argembeau, 2013; Feng et al., 2015; Jenkins & Mitchell, 2011), formation of self-oriented motives (Van Overwalle, 2009), and memory and decision making (Euston, Gruber, & McNaughton, 2012). In social neuroscience, the anterior cingulate cortex has been related, amongst other processes, to social learning and empathy (Behrens, Hunt, Woolrich, & Rushworth, 2008; Lockwood, 2016; Lockwood et al., 2016), which might explain why studies have associated this region with gaining at the expense of others.

When gaining shared rewards (e.g., rewards for both oneself and charity), we hypothesized that at least two processes would be important: the (anticipated) rewarding feeling of giving to others (probably related to ventral striatum activity) and evaluating the consequences for the self (possibly related to medial prefrontal cortex [mPFC] activity). Prior studies on prosocial giving have not yet distinguished between these two processes when examining giving to charity.

Thus, overall there is some inconsistency in the literature regarding the role of the ventral striatum in vicarious gaining. Although rewards for the self consistently engage regions in the ventral striatum (Báez-Mendoza & Schultz, 2013; Berridge & Kringelbach, 2015; Delgado, 2007; Montague & Berns, 2002; Schultz, 2016; Sescousse et al., 2013), studies that have examined vicarious rewards have shown less consistent results. Thus, it is important to investigate more thoroughly the underlying processes that might determine whether or not vicarious gaining, an important component of prosocial giving (Morelli et al., 2015; Telzer, 2016), is associated with ventral striatum activity. To address this issue in the present study, we aimed to further unravel which processes are important for ventral striatum activation in vicarious gaining. Therefore, we used a new functional magnetic resonance imaging (fMRI) paradigm in which we could keep the interpersonal factors that influence vicarious gaining relatively stable. That is, we focused on vicarious gaining for a charity, which is a relatively stable distal “other.” Participants chose a charity from a list of ten big charities (see Kuss et al., 2013, for a similar approach), and subsequently performed a task in the MRI scanner in which they could earn extra money for themselves and for the chosen charity by making a random, two-option decision on each trial. The different conditions for these outcomes were presented in a format such that several combinations of gains were possible: gains just for the self, gains just for charity, shared gains for both charity and the self, and no gains for either party. The size of the outcomes was varied, both to keep participants engaged in the task and to distinguish between absolute and relative amounts of gain (Tabibnia, Satpute, & Lieberman, 2008). In this way, vicarious gains for charity could be contrasted with gaining only for oneself and gaining for both parties, the latter of which had not been studied before. We focused on the roles of the ventral striatum and the medial PFC on the basis of prior studies that have suggested that both have an important role in prosocial behavior and self-related motivations (Bellucci et al., 2017; Feng et al., 2015). Since the exact contributions of these regions are not well understood in the context of prosociality, we examined the relation between neural responses to vicarious gains for charity, self-reported enjoyment of gaining for charity, and subsequent donating behavior to charity (Kuss et al., 2013). In addition, we examined the role of individual differences in empathic concern and in self-reported enjoyment for charity gains, to investigate whether the magnitude of activation in ventral striatum activity is dependent on individual differences in these domains.

We expected ventral striatum activation to be higher when gaining for the self than when there was no gain, consistent with prior studies showing a role of the ventral striatum in reward processing (Haber & Knutson, 2010; Morelli et al., 2015; Schultz, 2016). In combination with research that suggests that prosocial behavior may feel rewarding (Dunn, Aknin, & Norton, 2014; Telzer, 2016), we expected gain situations in which both the self and charity benefited to result in more ventral striatum activation than situations in which only the self gained (Krill & Platek, 2012), and that charity gains would result in higher activity in the ventral striatum than would no gain (Kuss et al., 2013). In addition to expectations about the effects of different outcomes, we hypothesized that individual differences in empathic concern, which have been shown to relate to ventral striatum activity (Morelli, Rameson, & Lieberman, 2014), and differences in the perceived importance of the charity, which have been shown to affect the pleasure of giving (Aknin et al., 2013), would both be related to variations in the neural reactions to gaining for charity. Finally, we predicted that participants who showed higher vicarious responses in the ventral striatum when gaining for charity would also donate more to charity (Kuss et al., 2013).

## Method

### Participants and procedure

Thirty-one participants between the ages of 21 and 24 participated in this study (16 males, 15 females; *M* = 22.54 years, *SD* = 1.19). The participants were recruited from a participant database. The ethics commission board of the Leiden University Medical Center (LUMC) approved the study and its procedures. Written informed consent was obtained from all participants, who were also right-handed and had normal or corrected-to-normal vision. Participants were screened with questionnaires on three separate occasions (once by phone call, once by email, and once on the testing day) for MRI contra-indications and for (history of) neurological and/or psychiatric disorders. All anatomical MRI scans were reviewed by a radiologist. No anomalous findings were reported.

### Materials

#### COSY fMRI task

**Fig. 1:**
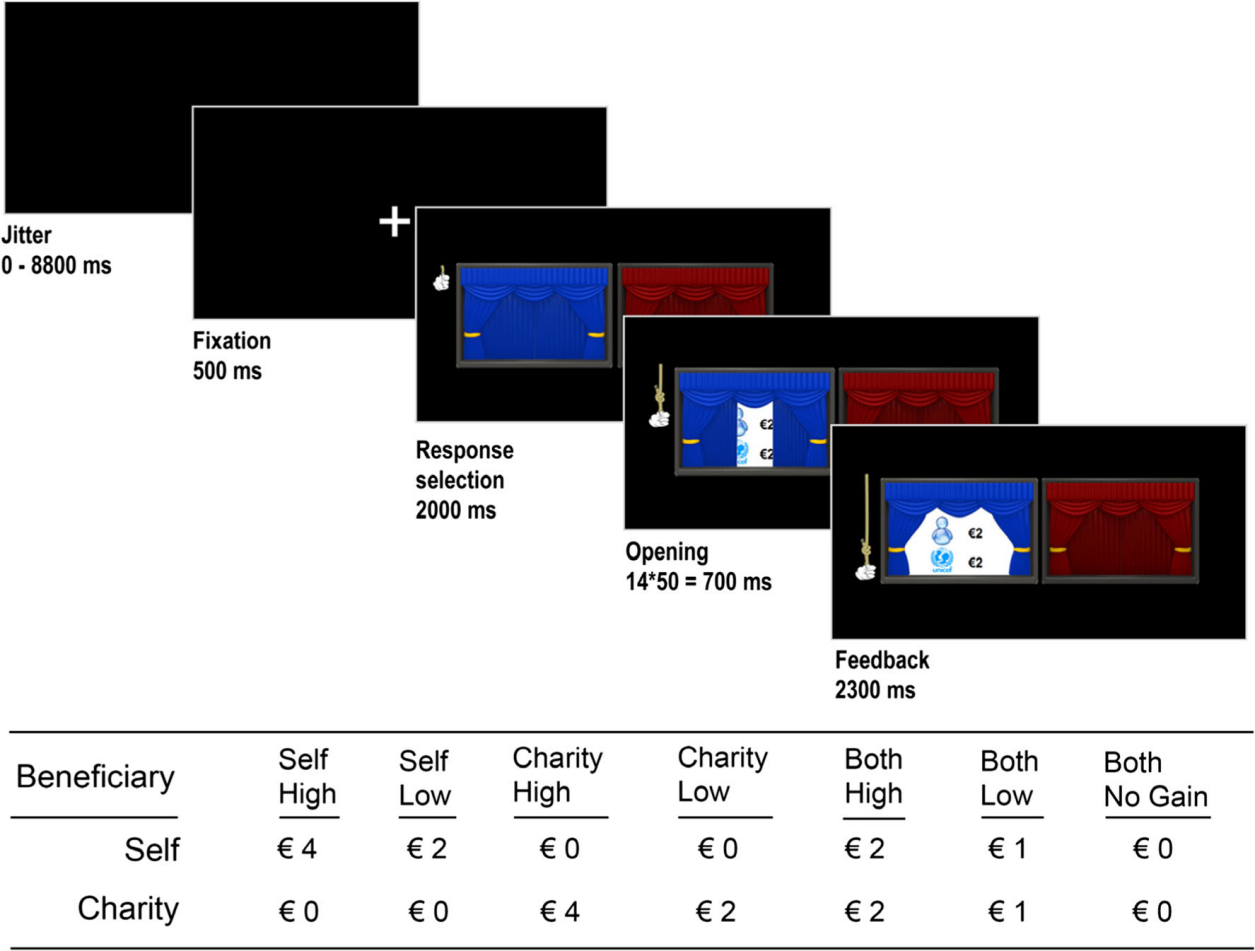
This figure shows the basic trial flow of the COSY task. Each trial started with a black screen with a jittered duration between 0 and 8,800 ms. Subsequently, a fixation cross was shown for 500 ms, followed by the response selection screen for 2,000 ms. After a response was made, a hand was shown onscreen for the remainder of the 2,000 ms. If a response was made, the next 14 screens showed a fluid animation of a hand pulling the curtain open, revealing the outcome (shown here = self €2, charity €2). The feedback remained onscreen for 2,300 ms. If participants failed to respond within the timeframe of response selection, a screen with the phrase “Too Late!” was instead shown for 3,000 ms. The possible outcomes are displayed in the table below the trial flow

To investigate responses to vicarious gains for charity, we developed a new fMRI task called the COSY (“charity or self yield”). In the COSY task, participants could earn money for themselves and for a previously self-chosen charity by deciding which of two curtains to open on every trial. After participants pressed a button, an onscreen hand indicated what option they had selected. Next, the chosen curtain opened in a fluid animation (14 frames presented for 50 ms each), with the outcomes being fully visible from the seventh frame onward. The overall outcome was a division of either €4 between parties or €2 between parties. In the case of a division of €4 (high stakes), this could result in the following outcomes: SelfHigh [€4 self, €0 charity], CharityHigh [€0 self, €4 charity], or BothHigh [both €2]. In the case of a division of €2 (low stakes), this could result in the following outcomes: SelfLow [€2 self, €0 charity], CharityLow [€0 self, €2 charity], or BothLow [both €1]. In both conditions, these outcomes were contrasted against a zero-gain baseline, BothNoGain [both €0]. The two stakes were used both to keep participants engaged in the task and to control for magnitude when examining the effects of mutual gain (Tabibnia et al., 2008). A black jitter screen (0–8,800 ms) was presented after the outcome presentation, marking the end of a trial (see Fig. 1 for a graphical representation of the trial flow and the outcome conditions).

Before participants played the COSY task in the scanner, they were given instructions for the fMRI task on a laptop. They were instructed which buttons to press during the fMRI task and played five randomly selected mock trials to get some experience with the short (2,000-ms) timeframe in which to make a response. Participants were not given any information about whether or not there were differences between the curtains or about the outcome distribution. Unknown to the participant, every outcome condition occurred 15 times during the task. This resulted in a total of 105 trials. The outcomes in the task were presented randomly to the participant, though the order of trials was optimized for our design using the program Optseq2 (Dale, 1999). The task consisted of two blocks, of 50 and 55 trials, respectively. Each block lasted approximately 6 min. At the end of the research day, participants received extra money based on the average of three random outcomes (ranging from €1 to €3 for both self and charity) and were informed of this beforehand.

### Questionnaires

#### COSY manipulation checks

After the fMRI session, participants answered several questions about the COSY task. First, to obtain a subjective measure of the enjoyment when gaining for charity, we asked how the different outcome conditions felt, to be answered on a scale from 1 = *did not feel good at all* and 7 = *felt very good*. Second, we asked whether participants thought they could influence the outcome and why they thought this was or was not the case. Finally, we asked participants how important they rated the charity, how well they knew what the charity stood for, and whether or not they normally donated to this charity in daily life. All questions were answered on 7-point Likert scales.

#### Empathy and perspective taking

To investigate individual differences in empathy and perspective taking, we used the Empathy and Perspective-Taking subscales of the Interpersonal Reactivity Index (IRI) questionnaire (Davis, 1980; Hawk et al., 2013). Both subscales were reliable, with Cronbach’s alpha values, respectively, of .72 and .79.

#### Behavioral donating task

In the exit interview after the fMRI session, participants played a one-shot Dictator Game for themselves and charity. Specifically, participants were given the choice to distribute 600 valuable coins (that were worth extra participant money) between themselves and the charity of their choice, by selecting one of seven possible divisions on a scale (1 = 600 for self, 0 for charity; 2 = 500 for self, 100 for charity; 3 = 400 for self, 200 for charity; 4 = 300 for self, 300 for charity; 5 = 200 for self, 400 for charity; 6 = 100 for self, 500 for charity; and 7 = 0 for self, 600 for charity). To prevent socially desirable behavior, it was stressed that the distribution chosen would remain completely anonymous. To ensure full anonymity, neither the participant nor the experimenter could see the outcomes of the donation task on the research day.

#### MRI data acquisition

MRI data were acquired using a Philips 3.0-T scanner with a standard whole-head coil attached. For the functional MRI scans, we used T2*-weighted echo-planar imaging (TR = 2.2 s, TE = 30 ms, FOV: 220 mm, 80 × 80 matrix, 2.75 mm in-plane resolution). The functional scans consisted of two runs with 175 and 169 volumes, respectively. Participants were able to see the screen through a mirror that was attached to the head coil. The functional task lasted for about 13 min in total. In addition to the fMRI sequences, we collected structural images for anatomical reference (high-resolution 3-D T1), TR = 9.751 ms, TE = 4.59 ms, FOV = 224 × 168 × 177 mm. Participants’ head movements were restricted by using foam triangles to limit the available space in the coil.

### MRI data analyses

#### Preprocessing

We used the software package SPM8 (Wellcome Trust Centre for Neuroimaging, London) to preprocess and analyze all MRI data. For preprocessing, we first corrected all MRI images for motion and slice timing acquisition, and consequently spatially normalized the functional scans to T1 templates. Then, all volumes were resampled to voxels of 3×3×3 millimeters. We based our templates on the MNI305 stereotaxic space (Cocosco, Kollokian, Kwan, & Evans, 1997). Finally, we used an isotropic Gaussian kernel (6-mm full width at half maximum) to spatially smooth the data.

#### fMRI analysis

To calculate the relevant contrasts, we modeled the fMRI time series convolved with the hemodynamic response function with events that corresponded to both the decision and outcome phases of a trial. For the outcome phase specifically, the events of interest that we modeled were the outcome conditions “SelfHigh,” “SelfLow,” “CharityHigh,” “CharityLow,” “BothHigh,” “BothLow,” and “BothNoGain.” These events were time-locked, with zero duration, to the exact moment that participants were able to see the outcome: the seventh frame of the curtain-opening animation. Trials with no response from the participants were coded as “Missing” and modeled separately as invalid trials, and they were not included in further contrasts. For the seven outcome conditions mentioned above, we also modeled the respective decision phases, with variable durations corresponding to participants’ reaction times. All modeled events were added as regressors in a general linear model, along with motion regressors, a basic set of cosine functions that high-pass filtered the data, and a covariate for session effects. The least squares parameter estimates of height of the best-fitting canonical hemodynamic response function for each condition were used in pairwise contrasts. The resulting contrast images, computed on a subject-by-subject basis, were submitted to random-effect group analyses. To test the hypotheses of this study, we tested three contrasts using second-level whole-brain group comparisons: *self-gain versus both-gain*, *self-gain versus charity-gain*, and *both-gain versus charity-gain*. No specific contrasts were made for magnitude of gain.

#### fMRI region-of-interest analysis

To further investigate the neural effects of vicarious gaining for charity in relation to magnitude of gain, we performed region-of-interest (ROI) analyses using the Marsbar toolbox (Brett, Anton, Valabregue, & Poline, 2002). We performed our analyses on a predefined anatomical ROI (Braams, van Duijvenvoorde, Peper, & Crone, 2015) of the left and right nucleus accumbens (nAcc), a part of the ventral striatum, which was extracted from the Harvard–Oxford subcortical atlas and thresholded at 40%. The mask consisted of 28 voxels for the left nAcc (coordinates left: *x* = – 9.57, *y* = 11.70, *z* = – 7.10) and 26 voxels for the right nAcc (coordinates right: *x* = 9.45, *y* = 12.60, *z* = – 6.69). We extracted parameter estimates for the ROI analyses. Given that none of the results showed differences between hemispheres, all the analyses were performed by collapsing across the left and right hemispheres.

## Results

### Behavioral results

#### COSY manipulation checks

Participants rated on a scale from 1 to 7 how much they had enjoyed winning different magnitudes of money (€1, €2, and €4) for themselves and charity. A 3 (magnitude) × 2 (beneficiary: self or charity) analysis of variance (ANOVA) resulted in a main effect of magnitude [*F*(2, 60) = 37.77, *p* < .001, *η*_p_^2^ = .62]. Follow-up analyses confirmed that €4 magnitudes were rated as more enjoyable than €2 magnitudes [*F*(1, 30) = 23.03, *p* < .001, *g* = .65], and €2 magnitudes were rated higher than €1 magnitudes [*F*(1, 30) = 34.01, *p* < .001, *g* = .61]. There was no main effect or interaction with charity, suggesting that winning for oneself (€1, *M* = 4.71; €2, *M* = 5.42; and €4, *M* = 6.13) and winning for charity (€1, *M* = 5.26; €2, *M* = 5.68; and €4, *M* = 6.00) were rated as equally pleasurable. As can be seen in Table 1, self-reported enjoyment when winning for oneself and self-reported enjoyment when winning for charity were not correlated.

**Table 1:** Correlations between IRI scores; COSY outcome enjoyment ratings for self, charity, and both; importance; knowledge; daily-life donating; and donation behavior

	IRI_PT	Self Gain	Char. Gain	Both Gain	Importance	Knowledge	Daily-Life Donating	Donation Behavior
IRI_EC	.47^**^	– .13	.44^*^	– .27	.38^*^	.47^**^	.24	.54^**^
IRI_PT		– .18	.07	– .15	– .05	.38^*^	.34	.13
Self gain			.22	– .23	– .06	.18	.03	– .41^*^
Char. gain				– .18	.42^*^	.33	.43^*^	.30
Both gain					– .10	– .23	– .20	– .14
Importance						.09	.21	.61^**^
Knowledge							.35	– .05
Daily-life donating								.08

^*^p < .05, two-tailed. ^**^p < .01, two-tailed

All possible charities were chosen at least once by the participants. The perceived importance of the chosen charity was relatively high for all participants (*M* = 5.58, *SD* = .91). Knowledge of the charity was more variable (*M* = 4.56, *SD* = 1.30). There were no correlations between the perceived importance of the charity and knowledge about the activities of the charity (all *p*s > .12).

Participants were asked whether they thought they could influence the outcome of the task (multiple choice: no/sometimes/yes). One of the participants answered “yes” to this question, 22 answered “no,” and eight answered “sometimes.” We reanalyzed the data while excluding the nine participants who provided the “yes” and “sometimes” answers, but this did not change the results. Therefore, we report the data from all participants.

Finally, to explore the impact of the various included individual difference measures on enjoyment for self-gains, charity-gains, and both-gains, we conducted regression analyses with enjoyment for the three beneficiaries (self-enjoyment, charity-enjoyment, and both-enjoyment) as respective dependent variables, and empathic concern, perspective taking, importance of charity, knowledge of charity, daily-life donating, and donation behavior as independent variables in separate analyses. First, for the regression analyses, none of the independent variables were significant predictors of self-enjoyment (all *p*s > .34). Second, for the regression analysis on charity enjoyment, empathic concern (*β* = .43, *p* = .014), importance of the charity (*β* = .42, *p* = .017), and daily-life donating (*β* = .43, *p* = .015) were significant positive predictors of charity enjoyment. Perspective taking and knowledge about the charity were not significant (both *p*s > .06). Third, with respect to the regression for both-enjoyment, none of the individual predictors were significant (all *p*s > .12) (see also Table 1).

#### Behavioral donating task

Participants donated on average 280 of the 600 coins to charity (*SD* = 164.8) in the one-shot donation Dictator Game. Donating behavior was normally distributed, with a skewness of .36 (*SE* = .42) and a kurtosis of .19 (*SE* = .82). Donating behavior was positively correlated to the IRI-EC subscale [*r*(31) = .54, *p* < .001], negatively correlated to how much participants enjoyed gaining for the self [*r*(31) = – .41, *p* = .021], and positively correlated to the self-reported ratings of the subjective importance of the charity [*r*(31) = .61, *p* < .001]. No other significant correlations were found with self-reported daily-life donation behavior.

To explore the impact of the individual difference measures on donation behavior, we conducted a regression analysis with donation behavior as the dependent variable and empathic concern, perspective taking, importance of the charity, knowledge of the charity, daily-life donating, and donation behavior as independent variables. Of the independent variables, empathic concern, importance of the charity, and daily-life donating were significant positive predictors. That is, the higher were empathic concern (*β* = .54, *p* = .002), charity importance (*β* = .61, *p* > .001), and daily-life donating (*β* = .43, *p* = .015), the more participants donated to charity in the one-shot donation Dictator Game. In addition, self-enjoyment negatively predicted donation behavior, with higher self-enjoyment (*β* = – .41, *p* = .021) being related to less donations (see also Table 1).

#### Empathy and perspective taking

Scores on the IRI Empathic Concern (IRI-EC) subscale ranged from 1.67 to 4.33, with *M* = 3.23 (*SD* = .63), and scores were approximately normally distributed, with skewness and kurtosis values between – 2 and 2 (Field, 2009; Gravetter & Wallnau, 2014); skewness = – .33 (*SE* = .42) and kurtosis = .22 (*SE* = .82). Scores on the Perspective Taking subscale (IRI-PT) ranged from 2.33 to 5, with *M* = 3.85 (*SD* = 0.63), a skewness of – .12 (*SE* = .42), and a kurtosis of – .06 (*SE* = .82).

### Neural activity

The results of the fMRI analyses are presented in two sections. First, we computed whole-brain contrasts for the three conditions by collapsing across magnitudes, and second, we tested the effects of condition and magnitude using ROI analyses of the nAcc (a region in the ventral striatum).

#### Whole-brain analyses

**Fig. 2:**
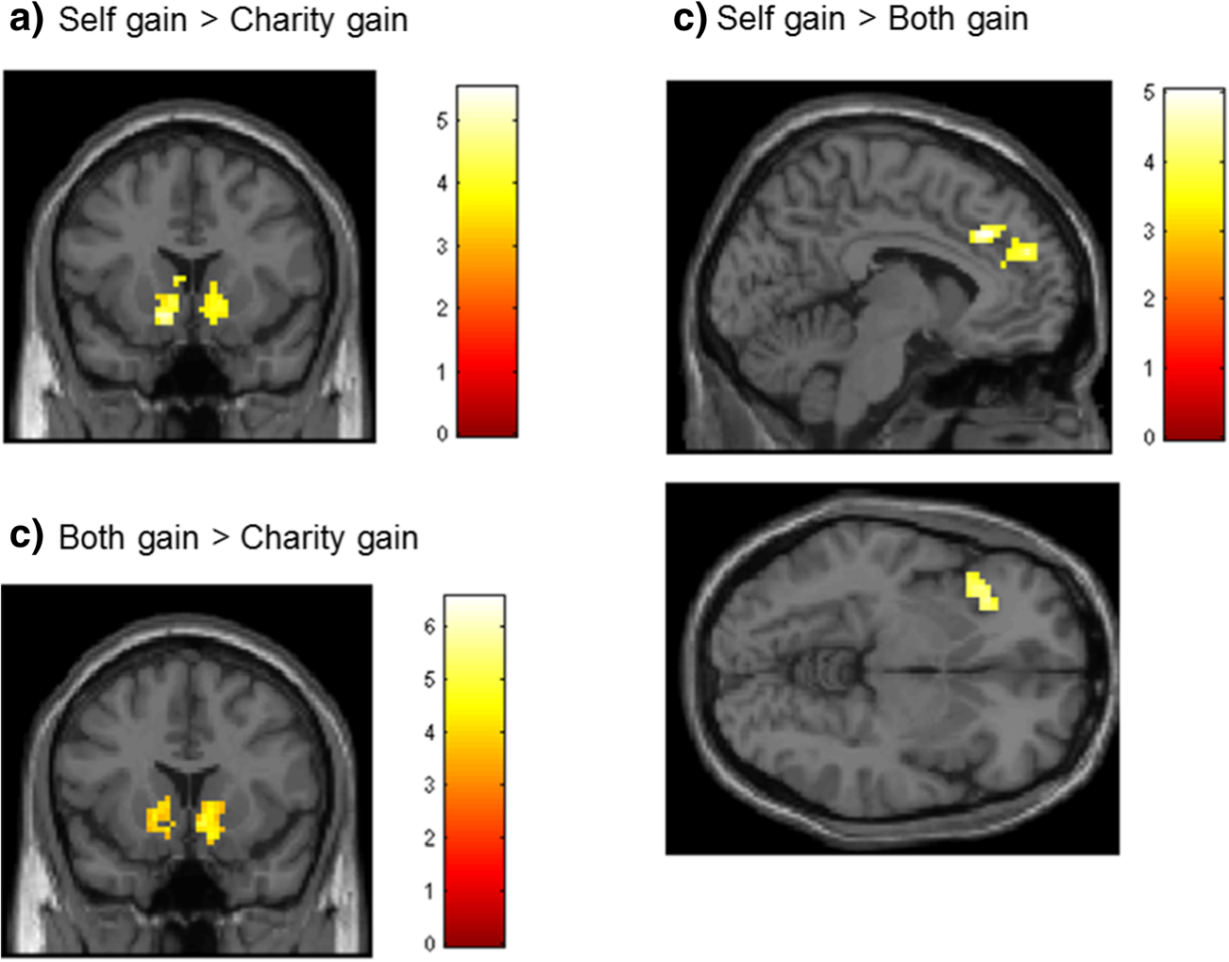
(A) Whole-brain results for the contrast Self-gain > Charity-gain, and the regions of interest based on this contrast. (B) Whole-brain results for the contrast Both-gain > Charity-gain, and the regions of interest based on this contrast. (C) Whole-brain results for the contrast Self-gain > Both-gain. All contrasts were family-wise error-corrected at *p* = .05

Since we expected that gaining for oneself would lead to more activation than vicarious gaining for charity, we first tested the contrast Self-gain > Charity-gain and the reversed contrast at the whole-brain level. Self-gain > Charity-gain resulted in bilateral activation in the ventral striatum (see Fig. 2 and Table 2). The reversed contrast (Charity-gain > Self-gain) did not result in significant activation.

**Table 2:** Coordinates for contrasts self-gain versus both-gain, self-gain versus charity-gain, and both-gain versus charity-gain, FDR cluster corrected (initial threshold *p* < .001)

Contrast	Region	T	K	*x*	*y*	*z*
*Self-gain* >	Right caudate	5.52	144	12	5	– 11
*Charity-gain*	Right putamen	4.73		18	20	– 8
	Right caudate	4.50		9	11	– 11
	Right insula	4.37		27	26	– 5
	Right insula	4.29		42	20	– 8
	Right caudate	3.74		9	17	– 11
	Left caudate	5.27	166	– 12	11	– 11
	Right thalamus	5.01		3	– 4	4
	Left caudate	4.78		– 9	11	– 2
	Right thalamus	4.77		3	– 10	7
	Left caudate	4.23		– 5	14	– 4
*Self-gain* >	Supplementary motor area	5.02	96	9	14	61
*Both-gain*	Right frontal superior gyrus	3.89		12	5	73
	Right frontal superior gyrus	3.47		18	2	57
	Left middle cingulum	4.89	105	– 9	29	34
	Right middle cingulum	4.77		12	29	34
	Right middle cingulum	4.28		15	23	31
	Left frontal superior gyrus	4.02		– 24	41	37
	Left frontal middle gyrus	3.93		– 27	47	37
	Left frontal superior gyrus	3.88		– 21	38	34
	Left insula	4.60	71	– 30	23	– 2
	Left frontal inferior orbitofrontal gyrus	4.23		– 39	23	– 5
	Left frontal inferior triangularis	3.55		– 48	17	1
	Right insula	4.47	33	33	23	– 17
	Frontal superior medial gyrus	4.35	66	– 6	50	25
	Right frontal superior medial gyrus	3.97		3	53	22
	Left frontal superior medial gyrus	3.91		– 15	41	22
	Right frontal superior medial gyrus	3.50		9	56	34
*Both-gain* >	Right middle temporal gyrus	4.2	90	42	– 52	– 2
*Self-gain*	Right inferior temporal gyrus	4.71		45	– 46	– 11
	Right inferior temporal gyrus	4.51		54	– 55	– 5
*Both-gain* >	Right calcarine gyrus	6.54	276	12	– 91	7
*Charity-gain*	Right primary visual area	6.29		15	– 76	7
	Right lingual gyrus	5.03		24	– 57	4
	Right precuneus	4.33		12	– 52	13
	Cerebellar vermis	4.11		5	– 57	– 5
	Right lingual gyrus	3.52		9	– 51	7
	Right thalamus	5.43	247	3	– 10	4
	Right caudate	5.10		9	17	– 8
	Right olfactory gyrus	5.03		6	20	– 11
	Right caudate	5.03		12	11	– 11
	Right caudate	4.63		12	17	– 2
	Right gyrus rectus	4.38		12	23	– 14
	Left olfactory gyrus	4.33		– 3	23	– 5
	Left caudate	4.12		– 9	14	– 11
	Left olfactory gyrus	3.91		– 3	17	– 8
	Left temporal superior gyrus	5.07	115	– 45	– 10	– 2
	Left temporal superior gyrus	5.06		– 48	– 4	– 8
	Left insula	4.68		– 39	– 13	– 5
	Left precentral gyrus	4.31		– 51	– 1	22
	Left temporal superior gyrus	4.21		– 57	– 7	7
	Left temporal superior gyrus	4.16		– 54	– 7	– 2
	Left postcentral gyrus	4.08		– 57	– 10	16
	Left rolandic operculum	3.95		– 48	2	16
	Left temporal superior gyrus	3.92		– 63	– 7	4
	Left precentral gyrus	3.77		– 42	2	22
	Left frontal inferior operculum	3.56		– 51	8	13
	Right temporal superior gyrus	4.91	55	63	– 16	13
	Right temporal superior gyrus	4.54		55	– 19	7
	Left occipital superior gyrus	4.91	73	– 12	– 94	7
	Left occipital middle gyrus	3.79		– 30	– 82	4
	Left temporal middle gyrus	4.73	69	– 45	– 58	1
	Left occipital middle gyrus	4.55		– 42	– 64	4
	Left temporal middle gyrus	4.05		– 48	– 52	– 2
	Left lingual gyrus	3.73		– 21	– 73	4
	Left calcarine gyrus	4.48	46	– 6	– 73	16
	Left precuneus	3.72		– 12	– 58	16

The Automated Anatomical Labeling (AAL) atlas was used for labeling. For coordinates that were not defined in the AAL atlas, we reported the nearest defined region

To investigate the unique contribution of gains for oneself, as compared to gains for both oneself and charity, we tested the contrast Self-gain > Both-gain and the reversed contrast Both-gain > Self-gain. This resulted in increased activation in a set of brain regions including regions in the supplementary motor cortex (SMA), medial prefrontal cortex (right/medial frontal superior gyrus) extending into the anterior cingulate cortex (cingulum), and bilateral insula (see Table 2 for a full list of the activations). The reversed contrast only resulted in one cluster of activation in the posterior parietal cortex (see Table 2).

In the final contrast, to explore the unique contribution of charity gains as compared to gains for both charity and oneself, we tested Charity-gain > Both-gain and the reversed contrast. The contrast Charity-gain > Both-gain did not result in any activation clusters. The reversed contrast again resulted in bilateral activation in the ventral striatum (see Table 2).

#### ROI analyses

Next, we performed ROI analyses to examine our a priori hypotheses concerning the ventral striatum in more detail. In cases in which the assumption of sphericity was violated, we report Greenhouse–Geisser corrections.

**Fig. 3:**
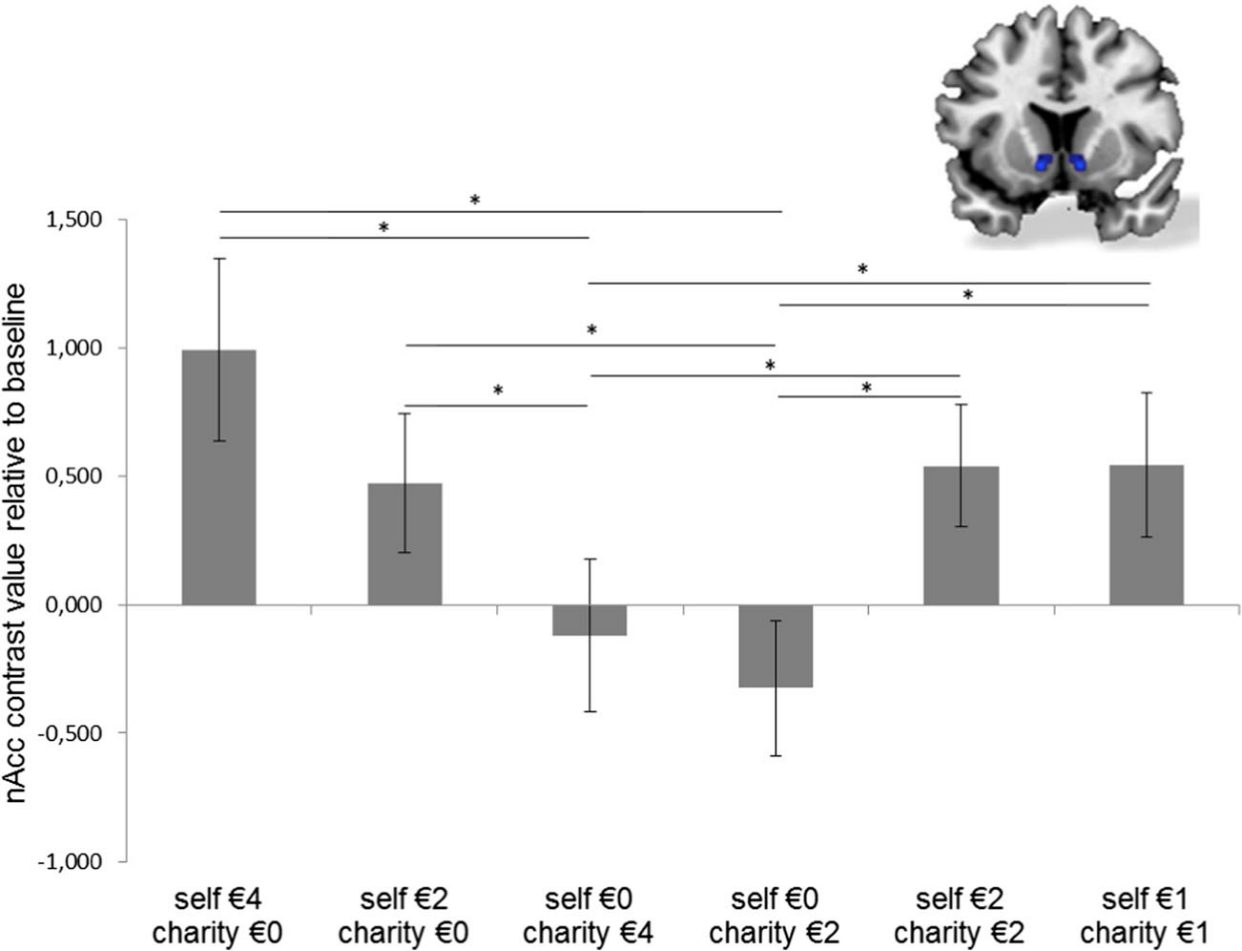
Whole-brain activity in the region of interest of bilateral nucleus accumbens (nAcc) in the ventral striatum. Different bars reflect the condition estimates minus the parameter estimate for the baseline condition. Error bars display 95% confidence intervals of the standard error of the mean. Asterisks reflect significance at *p* < .05

To disentangle the effects of beneficiary and outcome size, we conducted a repeated measures ANOVA with beneficiary (three levels: self, charity, both) and outcome size (two levels: high and low) as within-subjects factors. All parameter estimates in the repeated measures ANOVA were based on the different condition estimates minus the parameter estimate for the baseline condition (BothNoGain) (results are shown in Fig. 3).

The results yielded a main effect of beneficiary [*F*(1.58, 47.53) = 8.15, *p* < .001, *η*_p_^2^ = .21]. The main effect of outcome size and the interaction between beneficiary and outcome size were not significant [*F*(1, 30) = 2.44, *p* = .13, *η*_p_^2^ = .07, and *F*(1.87, 56.21) = 0.91, *p* = .41, *η*_p_^2^ = .029, respectively]. Post-hoc pairwise comparisons showed that the main effect of beneficiary was driven by significant differences between self-gain and both-gain versus charity-gain. More specifically, higher activity in the ventral striatum was found for self-gain (*M* = .73) than for charity-gain (*M* = – .22, *p* = .007), and also for both-gain (*M* = .54) than for charity-gain (*M* = – .22, *p* < .001). The differences between self-gain and both-gain were not significant.

### Relations between ventral striatum activation and individual differences

#### Enjoyment ratings

Next we investigated whether ventral striatum activation for self-gain, charity-gain, and both-gain were related to individual differences in enjoyment ratings.

**Fig. 4:**
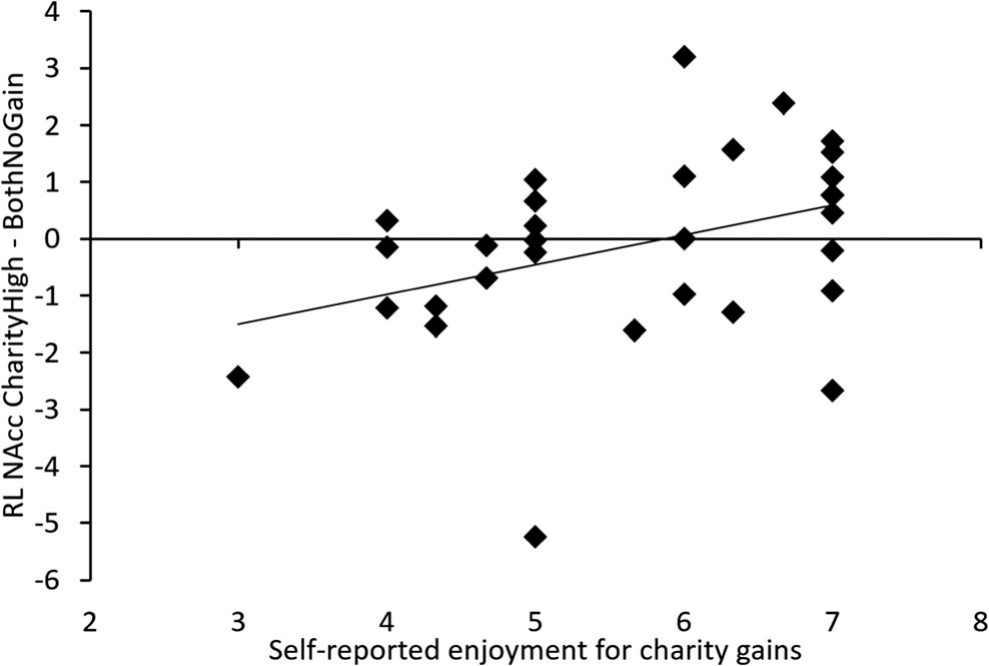
The *x*-axis shows the average of self-reported enjoyment for the charity gaining €1, €2, and €4. The *y*-axis shows neural activation in the ventral striatum, including the nucleus accumbens, in the CharityHigh > BothNoGain contrast

We correlated winning for charity (CharityHigh) relative to baseline (BothNoGain) with self-reported enjoyment of winning for charity. Higher enjoyment ratings for charity gains (averaged across €1, €2, and €4) were significantly correlated with higher ventral striatum activation for charity-gain [*r*(31) = .377, *p* = .037]. These results are shown in Fig. 4.

We found a similar pattern when examining enjoyment ratings when winning for oneself (averaged across €1, €2, and €4). Here, higher enjoyment ratings for self gains were significantly correlated with higher ventral striatum activation for self-gain [*r*(31) = .45, *p* = .01].

Interestingly, activation when gaining for both oneself and charity relative to baseline (BothHigh – BothNoGain) was positively correlated with enjoyment ratings of gaining for oneself [*r*(31) = .36, *p* = *.*046], but was not significantly correlated with enjoyment ratings of gaining for charity (*p* = .987).

Finally, to test whether the correlations between empathic concern and enjoyment of gaining for oneself were higher than the correlations between empathic concern and enjoyment of gaining for charity, we used the R “cocor” package (Diedenhofen & Musch, 2015). There were no significant differences between the correlations (all *p*s > .67).

#### Empathic concern and perspective taking

**Fig. 5:**
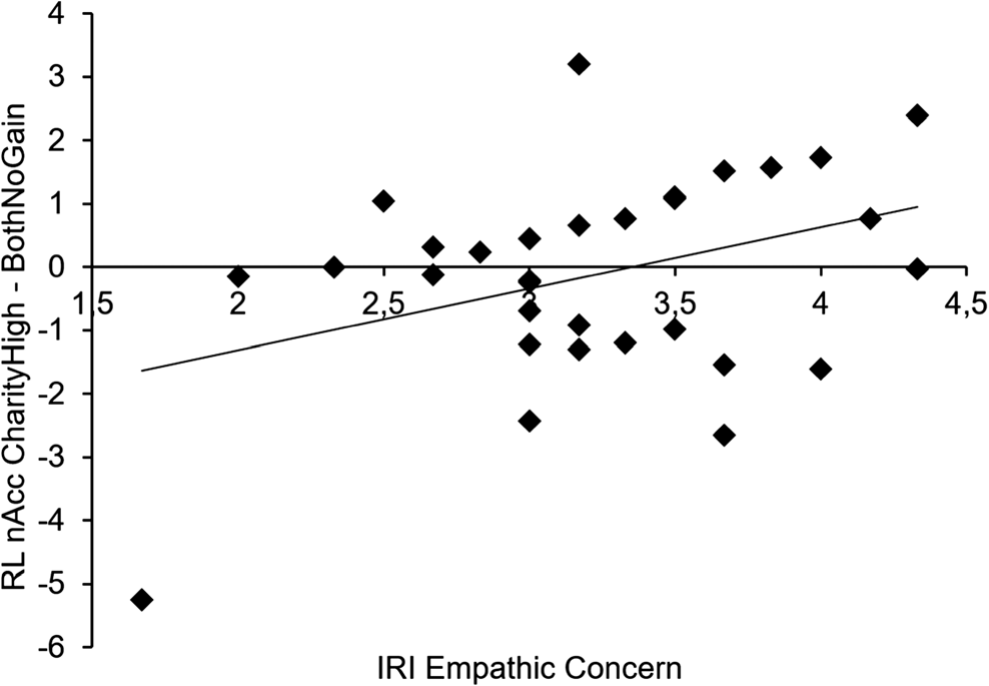
The *x*-axis shows scores on the IRI Empathic Concern subscale. The *y*-axis shows neural activation in bilateral nucleus accumbens (nAcc, a region in the ventral striatum) in the CharityHigh > BothNoGain contrast

Next, we tested whether ventral striatum activation for self-gain and for charity-gain (both relative to the baseline BothNoGain) were correlated with individual differences in empathic concern and perspective taking. We found a significant correlation between empathic concern and charity-gain [*r*(31) = .44, *p* = .014], but not between empathic concern and self-gain (*p* > .4). See Fig. 5 for a graphical representation of the relation between empathic concern and charity-gain. We found no significant correlations between perspective taking and ventral striatum activation (all *p*s > .4).

#### Donating behavior and importance ratings

To investigate whether actual donating behavior was related to ventral striatum activation for self and charity gains, we computed the correlations between neural activity in the nAcc ROI for self-gain and charity-gain and donating behavior. None of these correlations were significant (both *p*s > .5). Next, we computed the correlations between charity-gain conditions (CharityHigh and CharityLow) and self-reported ratings of the subjective importance of the charity. These correlation were also not significant (both *p*s > .2)

## Discussion

The goal of this study was to test neural responses in the ventral striatum during the processing of vicarious rewards for charity, when pitted against both self-gain and mutual gain. Consistent with prior research (Braams et al., 2014a; Harbaugh et al., 2007), there was robust activity in the ventral striatum for self-gains, independent of whether gains were obtained for oneself only, or for both self and charity. However, no striatum activity was observed for vicarious gains for charity. Interestingly, the neural responses to gains for charity were dependent on individual differences in self-reported enjoyment of gaining for charity and empathic concern.

### Gaining and the striatum

Studies have consistently shown that the ventral striatum is engaged when gaining for oneself (Berridge & Kringelbach, 2015; Braams, Peters, et al., 2014b; Delgado, 2007; Montague & Berns, 2002; Morelli et al., 2015; Schultz, 2016; Sescousse et al., 2013). In this study, striatum activity for self-gains was correlated with self-reported enjoyment of these gains, providing further evidence that striatum activity may reflect the subjective pleasure of receiving a reward (Báez-Mendoza & Schultz, 2013). We hypothesized that this subjective feeling of pleasure might be larger when gaining for two parties (charity and self) than when gaining only for oneself, but we did not find evidence for this hypothesis in the present study. That is, the ventral striatum was most strongly activated when gaining for the self only, as compared to when gaining for charity only or when gaining for both charity and oneself. Although ventral striatum activation was observed in the both-gain condition, this activation was possibly related to the reward gained for the self rather than to the mutual beneficial outcome, which is supported by its significant correlation with self-reported enjoyment of self-gain but not with charity-gain. Possibly, the rewarding effects related to a mutual beneficial outcome are only observed in active tasks in which participants work toward a goal together, such as in a prisoner’s dilemma type tasks (Krill & Platek, 2012; Rilling et al., 2004; Sun et al., 2016). Future studies should examine in more detail the effect of relative gain for the self and for another, and specifically should investigate under what circumstances mutually beneficial outcomes are experienced as being more rewarding than outcomes that are only beneficial for oneself.

The finding that less ventral striatum activation was present for charity-gain than for both-gain or self-gain conditions was contrary to our expectations. An earlier study examining vicarious gains for charity (Harbaugh et al., 2007) had found evidence for a “warm glow” effect, although this discrepancy could have been due to the smaller gains for charity in the present study (max €4, as compared to a max of ~ €38 in Harbaugh et al., 2007). However, several studies on vicarious-reward processing using amounts of money comparable to our paradigm have found that participants showed similar activity in the ventral striatum when gaining for friends (Braams, Güroǧlu, et al., 2014a) and mothers (Braams & Crone, 2017), as compared to the self, and when gaining for in-group as compared to out-group members (Hackel, Zaki, & Van Bavel, 2017). Possibly this vicarious reward response varies as a function of the strength of the personal connection to the target, which would explain why striatum activity is consistently observed for close others such as friends or family members, but not for more distant others such as charities, regardless of the possible need of the other party. This could be one possible explanation for why it can be difficult to engage people in society to perform prosocial behaviors such as giving to charity, and it should be more thoroughly investigated in future research.

Interestingly, even though self-gains led to more striatum activity than did charity gains (as compared to baseline), participants rated winning for self and for charity as being equally enjoyable. Perhaps these self-reported estimates of enjoyment obtained through questionnaires might have been influenced by social desirability, explaining the disjunction between neural measures of reward and self-report of reward.

Although there was no general striatum effect of gaining for charity, there were important individual differences. First, individuals who reported enjoying gaining for charity more also showed more ventral striatum activity for charity gains. Similarly, individuals with higher self-reported empathic concern also showed more ventral striatum activity for charity gains and donated more to charity afterward. Both results are consistent with studies showing that the rewarding feeling of giving to close others can be dependent on individual differences, such as in the perceived importance of the other and in relationship strength (Braams et al., 2014a; Inagaki et al., 2016; Kuss et al., 2013; Telzer et al., 2013). Our results extend on these findings by showing that individual differences in empathic concern can also predict the rewarding feeling of giving to distant others, such as charities. However, we examined reward processing after vicarious gains in a very specific setting—that is, in a paradigm without control over the outcomes and in which the gains for a charity were not costly for the participants. Therefore, it should be noted that future research will be needed to unravel whether our results are generalizable to situations in which participants have more or full control over the outcome, or to situations in which participants have to give up resources to benefit the charity. On the other hand, the significant relations between donating behavior and empathy and between neural activation in the ventral striatum and empathy suggest that the underlying processes, both in vicarious gaining and in real-life donating behavior, might be similar, although more research will be necessary to investigate this.

### Gaining and the medial prefrontal cortex, anterior cingulate cortex, and insula

Besides activity in the ventral striatum, we also observed activity patterns that were greater for self-gain than for both-gain. That is, regions in the medial prefrontal cortex (medial frontal gyrus) extending into the anterior cingulate cortex (cingulum), as well as bilateral insula, were active when gaining more for the self, relative to gaining for both self and charity. The medial prefrontal cortex (PFC) is a heterogeneous region involved in many functions. For instance, prior studies have suggested that the medial PFC is important for a range of cognitive processes, such as memory and decision making (Euston et al., 2012), integrating social information (Van Overwalle, 2009), self-orientation, self-reflection (Feng et al., 2015; Jenkins & Mitchell, 2011; Zaki & Mitchell, 2011), and fairness considerations regarding the self (Civai, Miniussi, & Rumiati, 2014). In the present study, it could be that medial PFC activation reflected self-relevance, since one of the prime differences between the self-gain and both-gain conditions was that the self condition was more self-relevant (in which the participant gained money and nothing happened for charity) than the both-condition (in which both parties gained money). However, the peak activation was more posterior in our study than in these studies. It should also be noted that medial PFC and bilateral insula were not significantly more active for self-gain than for charity gain, making it unlikely that self-oriented processes were the only explanation for the medial PFC and insula activity.

In addition, we considered fairness considerations as a possible process that might underlie differences in medial PFC and insula activation between the self- and both-gain conditions. Since the stakes were divided equally in the “both” conditions and unequally in the “self” conditions, this inequality might have triggered fairness considerations. Furthermore, it is possible that activation in the medial PFC and the insula together might reflect feelings of shame or guilt (Bastin, Harrison, Davey, Moll, & Whittle, 2016; Michl et al., 2014). That is, participants might feel more guilty or ashamed when they gain money for themselves only than when they gain money for a charity or for both themselves and a charity. Neither explanation accounts for the absence of medial PFC activation when comparing self-gain to charity-gain; therefore, future research will be necessary in order to understand the exact roles of medial PFC and insula when comparing gains for oneself only versus gains for others.

In addition, the increased activation that we found in medial PFC for self-gain as compared to both-gain extended into the anterior cingulate sulcus (ACCs). Activation in this region has previously been associated with—among other processes—information processing when learning in nonsocial contexts (Apps, Rushworth, & Chang, 2016), personal pain, and empathy for others experiencing pain (Lockwood, 2016). Whereas it is unlikely to be the case that the stronger activation in self-gain than in both-gain reflects pain for the participant (since self-gain is objectively better for the participant), it might be the case that the activation in the ACCs in this contrast reflects activation related to learning. That is, even though participants objectively could not learn in the present study, since trials were presented in a random sequence, they might have attempted to find patterns in order to maximize self-gain.

### Limitations and future directions

When interpreting our results, several limitations need to be taken into account. Since our paradigm was jittered between trials but not between the decision phase and the outcome phase within a trial, we cannot temporally distinguish between these phases. However, even though we cannot temporally distinguish, we can still statistically distinguish the decision and outcome phases. That is, since the decision phase was identical in each of our conditions, any activation associated with the decision phase would be washed out across contrast conditions. Thus, any significant activations found in the contrasts had to be the result of a systematic difference between outcome conditions. Second, an interesting direction for future research would be to use prediction error models to investigate how participants react. For our present paradigm, a classical prediction-error model would not be the best fit, because one of our intentions was to develop a task without a real choice for the participant, to ensure that we would have enough trials in each payoff condition. Considering the answers to the control question gauging whether participants thought they were able to influence the outcome, it seems that this was effective. Therefore, it seems unlikely that participants built up expectations related to their choices, making our paradigm unsuitable for analyzing prediction errors.

## Conclusions

To conclude, this study showed that self-gains were consistently related to activity in the ventral striatum, replicating earlier studies (Berridge & Kringelbach, 2015; Braams, Peters, et al., 2014b; Delgado, 2007; Montague & Berns, 2002; Morelli et al., 2015; Schultz, 2016; Sescousse et al., 2013). Furthermore, although we found no rewarding effect (“warm glow”) of vicarious gaining for charity on a group level, we showed that this activity for vicarious charity gains was related to individual differences in empathy and self-reported enjoyment for charity gains. These findings support earlier literature (Braams, Peters, et al., 2014b; Inagaki et al., 2016; Telzer et al., 2013) suggesting that the strength of the relationship to the target is important for the “warm glow” effect to be found in vicarious gaining settings. Some studies have shown that reputation building may indeed increase activity in the ventral striatum in conditions of reciprocity, supporting this idea (Mobbs et al., 2015; Phan, Sripada, Angstadt, & McCabe, 2010). To further this line of research, future studies could examine these social context factors on charity giving in more detail, by investigating the role of relationships to specific charities in explaining the rewarding feeling of vicarious gaining.

### Author note

This work was supported by The Netherlands Organization for Scientific Research (NWO-VICI 453-14-001 E.A.C.). The authors thank all participants for their participation. We also thank Laura van der Aar, Renske van der Cruijsen, Miranda Lutz-Landesbergen, Marjolein Wille, and Isabel Zwaan for their help with the data collection.
